# Unravelling the complexity of domestication: a case study using morphometrics and ancient DNA analyses of archaeological pigs from Romania

**DOI:** 10.1098/rstb.2013.0616

**Published:** 2015-01-19

**Authors:** Allowen Evin, Linus Girdland Flink, Adrian Bălăşescu, Dragomir Popovici, Radian Andreescu, Douglas Bailey, Pavel Mirea, Cătălin Lazăr, Adina Boroneanţ, Clive Bonsall, Una Strand Vidarsdottir, Stéphanie Brehard, Anne Tresset, Thomas Cucchi, Greger Larson, Keith Dobney

**Affiliations:** 1 Department of Archaeology, University of Aberdeen, St Mary's Building, Elphinstone Road, Aberdeen AB24 3FX, UK; 2 CNRS-Muséum National d'Histoire Naturelle, UMR 7209, Archéozoologie, archéobotanique, 55 rue Buffon, 75005 Paris, France; 3 Durham Evolution and Ancient DNA, Department of Archaeology, University of Durham, South Road, Durham DH1 3LE, UK; 4 Department of Anthropology, University of Durham, South Road, Durham DH1 3LE, UK; 5 National History Museum of Romania, Calea Victoriei no. 12, District 3, 030026 Bucharest, Romania; 6 Department of Anthropology, College of Liberal and Creative Arts, San Francisco State University, 1600 Holloway Avenue, Science 377, San Francisco, CA 94132, USA; 7 Teleorman County Museum, str. 1848, no. 1, 140033 Alexandria, Romania; 8 Institute of Archaeology ‘Vasile Pârvan’ of the Romanian Academy, 11 Henri Coandă St., Bucharest, Romania; 9 School of History, Classics and Archaeology, University of Edinburgh, William Robertson Wing, Old Medical School, Teviot Place, Edinburgh EH8 9AG, UK

**Keywords:** domestication, neolithization, morphometrics, ancient DNA, *Sus scrofa*

## Abstract

Current evidence suggests that pigs were first domesticated in Eastern Anatolia during the ninth millennium cal BC before dispersing into Europe with Early Neolithic farmers from the beginning of the seventh millennium. Recent ancient DNA (aDNA) research also indicates the incorporation of European wild boar into domestic stock during the Neolithization process. In order to establish the timing of the arrival of domestic pigs into Europe, and to test hypotheses regarding the role European wild boar played in the domestication process, we combined a geometric morphometric analysis (allowing us to combine tooth size and shape) of 449 Romanian ancient teeth with aDNA analysis. Our results firstly substantiate claims that the first domestic pigs in Romania possessed the same mtDNA signatures found in Neolithic pigs in west and central Anatolia. Second, we identified a significant proportion of individuals with large molars whose tooth shape matched that of archaeological (likely) domestic pigs. These large ‘domestic shape’ specimens were present from the outset of the Romanian Neolithic (6100–5500 cal BC) through to later prehistory, suggesting a long history of admixture between introduced domestic pigs and local wild boar. Finally, we confirmed a turnover in mitochondrial lineages found in domestic pigs, possibly coincident with human migration into Anatolia and the Levant that occurred in later prehistory.

## 1. Introduction

The study of animal domestication has a long history that is intimately linked to wider research into the origins and subsequent spread of early farmers. Hypotheses rooted in the idea of a single origin and subsequent spread from domestication centres compete with those that suggest multiple, geographically independent origins. Traditional zooarchaeological methods have thus far not been able to conclusively distinguish between these scenarios. This is especially true for the progenitor of domestic pigs (*Sus scrofa*) whose extensive Old World distribution has made it difficult to pinpoint either where or how many times pigs were domesticated [1–6]. Through the generation of high-resolution datasets, new genetic and morphometric techniques are providing more powerful means of exploring the zooarchaeological record, making these questions more tractable.

The traditionally accepted model for pigs is that local wild boar populations were domesticated independently in Eastern Anatolia during the ninth millennium BC [1–3] and in central China during the seventh millennium BC [7,8]. Domestic pigs then dispersed outwards from these regions as part of the spread of Neolithic farming cultures. An assessment of mitochondrial variation in modern *Sus* populations across the Old World identified at least six geographically constrained genetic signatures shared by both local wild boar and domestic pigs, an observation that was initially used to support multiple (independent) domestication centres [9]. This study also found that modern European domestic pigs possessed the same mitochondrial signatures as European wild boar, suggesting that if Near Eastern pigs had been transported into Europe, they had left no mitochondrial genetic legacy in modern populations.

Subsequent ancient DNA (aDNA) studies [10,11] revealed a high frequency of a specific mtDNA haplotype (Y1) in ancient and modern Near Eastern *S. scrofa* and an absence of Y1 in both Mesolithic [10] and modern European wild boar [9]. Importantly, pig remains recovered from archaeological sites in Eastern Europe (dated to the mid-sixth millennium BC) primarily carried the Y1 haplotype identical to that found in both recent Near Eastern wild boar and Neolithic wild and domestic pigs from the Near East [11], corroborating the hypothesis that the first domestic pigs in Europe arrived from the Near East with Neolithic farmers.

Although the first domestic pigs in Europe carried a mitochondrial signature (Y1) endemic to Anatolia, numerous pigs from later prehistoric sites possessed mtDNA haplotypes identical to those associated with ancient and modern European wild boar (E1-A and E1-C). In addition, the Y1 haplotype was completely replaced in Europe by at least 3900 cal BC [10]. The limited power of mitochondrial datasets to discriminate between independent domestication and introgression, however, meant that the processes responsible for these observations remained uncertain.

Two recent reviews have suggested that the role of long-term (continuous) gene flow between wild and domestic animals in the development of the genetic and phenotypic constitutions of both past and present domestic animal populations has been underappreciated, and pigs are no exception [12,13]. Identifying complex scenarios involving dispersal and subsequent introgression between introduced domestic and local wild forms requires a combination of approaches, including both genetic and geometric morphometric (GMM) techniques that together possess sufficient resolving power to identify a continuum of states between wild and domestic forms. More specifically, while ancient DNA techniques are now commonly used to identify population shifts through time, GMM methods allow for the study of both size and shape changes over time and space, and have already been employed extensively to detect phenotypic changes in tooth morphology linked to pig domestication [8,11,14–17].

In this study, we focused on the Balkan Peninsula, a principal gateway through which Europe's first farmers migrated from western Anatolia and a key region in the early Neolithization of Europe [18–22]. The Neolithic cultures of Europe spread primarily along two main routes: by sea along the northern coastline of the Mediterranean, and by land, where colonization of the southeastern part of Europe followed the natural corridors of major river valleys such as the Vardar–Morava corridor, the Maritsa basin and the middle and lower Danube basins [6,23,24]. The richness of the Neolithic archaeology of the Balkan Peninsula and its geographical location makes the region of present-day Romania ideal for studying the Neolithization of Europe and for testing hypotheses about the introduction of the Neolithic farming package.

Bones and teeth of *S. scrofa* dating to the Early and Middle Neolithic are rare, not only in Romania [25,26], but also in numerous other early Neolithic cultures throughout Europe [27]. During the subsequent millennia, however, pig remains became more numerous, reflecting the growing importance of pigs as an economic resource. In Romania, this transition coincided with the Gumelniţa culture (Middle Chalcolithic: 4600–3600 cal BC), a period of cultural change, including increased social stratification and the development of copper metallurgy, tell sites and specialized husbandry practices [28].

Here, in order to assess the population history and relationship between domestic pigs introduced from the Near East and wild boar indigenous to Eastern Europe, we first used a novel identification protocol (electronic supplementary material, text and table S2) to determine the wild and domestic status of each archaeological tooth by assessing molar size and shape. This was achieved using GMM techniques on 449 archaeological pig teeth (from 377 individuals) including upper and lower second (M^2^ and M_2_) and third (M^3^ and M_3_) molars alongside 1064 West Palearctic specimens belonging to both modern wild and domestic pigs (detailed in references [14,29]). In addition, we obtained a phylogenetically informative *ca* 74 bp mtDNA fragment that captured a core set of variable nucleotide sites distinguishing the four major haplogroups (E1, E2, NE1 and NE2) previously identified among West Eurasian wild boar [9–11]. Ancient genetic signatures were obtained from 45 specimens (39 of which were also typed using GMM). These samples were collected from 18 Romanian archaeological sites (electronic supplementary material, figure 1S and text) ranging from the Mesolithic to the Iron Age (electronic supplementary material, table S1). We contrasted the GMM and aDNA results with previously published aDNA data from Romania [10] and lastly, obtained six direct accelerator mass spectrometry radiocarbon dates from key specimens (electronic supplementary material, text and table S3).

## 2. Results

**Figure 1.**
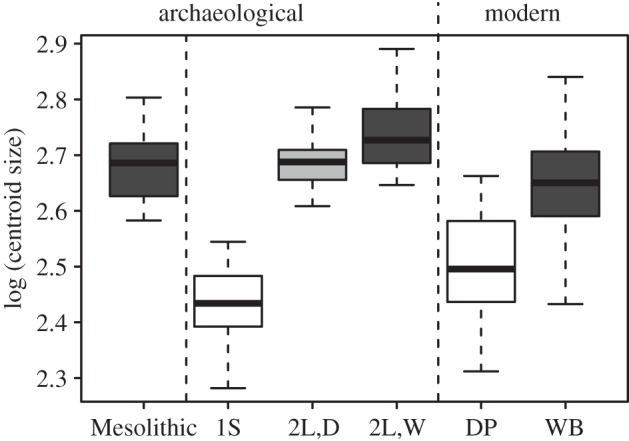
Boxplot of the log (centroid) size of the lower third molar. Specimens with small molars (group 1S) and with large molars both wild (2L,W) or domestic (2L,D) tooth shape were identified and compared with Mesolithic and modern wild boar (WB) and domestic pigs (DP).

The GMM size analysis revealed two groups: one consisting of individuals with small molars (group 1S), and another consisting of individuals with large molars (group 2L), both of which were present in all the archaeological periods (figure 1 and electronic supplementary material, table S1). When compared with both modern and Mesolithic specimens, group 1S individuals possessed significantly smaller molars than both the modern wild boar and all Mesolithic specimens used in this study. In addition, they were also smaller than all modern domestic pigs (a pattern consistent for all molars except M_2_; electronic supplementary material, table S5). Group 2L individuals possessed molars that were generally larger than modern wild boar, but similar in size to Mesolithic specimens, and always larger than modern domestic pigs (electronic supplementary material, table S5 and figure S2).

Of the factors known to impact the size of an organism (e.g. climate, insularity, sexual dimorphism and domestication), only the last two likely explain our data, because the two size groups occur contemporaneously and throughout all the studied time periods. Sexual dimorphism can be eliminated, because no clear size groupings for molar teeth can be seen in the modern wild boar populations analysed here. All previous zooarchaeological research has established the fundamental principle of size reduction from wild to domestic forms [30–35] in early animal domesticates. In fact, the lack of intermediate sized *S. scrofa* in Europe has been used as an argument for the direct introduction of domestic pigs and the lack of involvement of indigenous European wild boar in the domestication process [4]. Given the likelihood of gene flow between the introduced and native pig populations [12,13], however, we tested the expectation that all large pigs were in fact wild boar.

First, we assumed that individuals in group 1S can be used as a baseline reference for archaeological ‘domestic’ molar shape. Although we cannot exclude the possibility that this group includes some hybrid or feral individuals, this group as a whole most likely represents a population that had been domesticated for several thousand years. To generate a baseline for wild tooth shape, we grouped Mesolithic specimens (all of which were sampled from time periods prior to the introduction of domestic pigs from the Near East) from Romania, Switzerland [36] and France [37]. The inclusion of Mesolithic wild boar outside Romania (where our sample size was small) ensured that we maximized the shape variability associated with wild boar. In order to determine the wild or domestic status of all group 2L individuals, we compared them with the archaeological baseline domestic (group 1S) and wild (Mesolithic) datasets (see the electronic supplementary material, figure S3 for shape differences between these two groups).

The results of this analysis revealed that, though the majority (60%) of group 2L individuals possessed a wild tooth shape, a large proportion (40%) possessed a domestic shape (figure 1; electronic supplementary material, table S2 and text). Interestingly, those with a domestic tooth shape were on average smaller than those with a wild shape—a pattern consistent with the process of size reduction associated with the process of early domestication (electronic supplementary material, table S5).

**Figure 2.**
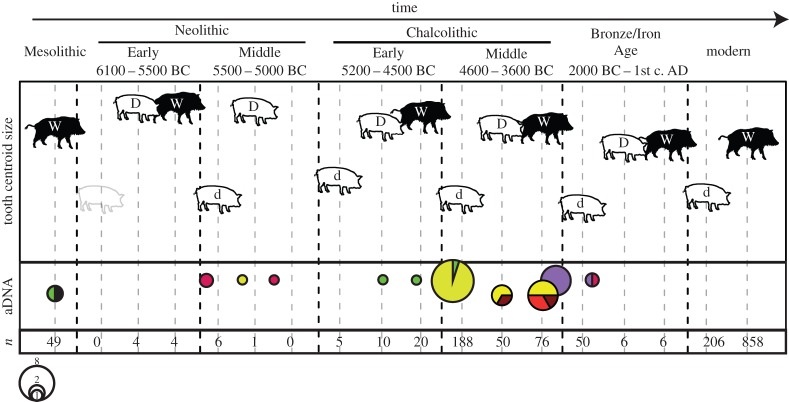
Evolution of tooth centroid size (M_2_) (*y*-axis) through time (*x*-axis) for small domestic pigs (d), large pigs with domestic tooth shape (D) and large pigs with wild tooth shape (W). The schematic (shadowed) small pig of the Early Neolithic represents the small domestic pigs present in Romania at this period based on traditional metrical data (humerus, [26]) but not present in our data. Also shown is the proportion of different haplotypes: yellow for Y1, maroon for AS1, red for E1-C, purple for E1-A, black for the new haplotype AS2 and green for Y2. Total sample size (*n*) including all four teeth for each category. The original figure showing data for all teeth is in electronic supplementary material, figure S2. The data were constrained within a chronological framework that consisted of six periods [48]: Mesolithic (8000–6100 cal BC), Early Neolithic (6100–5500 cal BC), Middle Neolithic (5500–5000 cal BC), Early Chalcolithic (5200–4500 cal BC), Middle Chalcolithic (4600–3600 cal BC; [28]) and a pooled phase combining Bronze and Iron Age (i.e. later prehistory; 2000 cal BC to first century AD).

Following the assessment of wild and domestic tooth shape, we tracked the temporal changes in both the molar size and shape (figure 2 and electronic supplementary material, figure S4). As previously described, group 2L specimens with both wild and domestic tooth shapes were present across all time periods (figure 2 and electronic supplementary material, table S1). Group 1S individuals were also present from the Middle Neolithic (5500–5000 cal BC) onwards.

The aDNA analysis revealed the presence of six haplotypes [10,11] (electronic supplementary material, figure S5) among the ancient Romanian pigs (electronic supplementary material, table S3): Y1, Y2, AS1, AS2, E1-A and E1-C. Mesolithic specimens carried haplotypes AS2 (*n* = 1) and Y2 (*n* = 1), which together with previously published Mesolithic data from Romania (two pigs carrying the E1-A and E1-C haplotypes, but see electronic supplementary material, table S3), suggest that this geographical region harboured more genetic variation than previously known [10]. Wild specimens (*n* = 6) from the Neolithic and Chalcolithic carried haplotypes E1-C and Y1, the latter likely the result of introgression from domestic stock brought to Europe from the Near East [12].

Group 1S specimens dating to the Neolithic and Chalcolithic (figure 2 and electronic supplementary material, figure S6) nearly exclusively (17/18) carried the Y1 haplotype (the sole exception possessed the Y2 haplotype). The group 2L pigs with domestic tooth shape (*n* = 6) dating to these same periods (that yielded DNA results), however, possessed a variety of haplotypes (*n* = 5; E1-C, E1-A, AS1, Y1 and Y2), suggesting significant diversity likely bolstered by a history of admixture with introduced domestic pigs.

When placed in a more detailed cultural–temporal context, we find that the earliest appearance of the Y1 haplotype in our Romanian dataset dates to the Middle Neolithic (Vădastra culture, directly dated to 5318–5206 cal BC/6260 ± 34 BP) at the site of Măgura–Buduiasca [38] and was carried by group 1S domestic pigs. The earliest genotyped group 2L specimen with a domestic tooth shape is from the Middle Neolithic context of Măgura–Buduiasca (directly dated to 5307–5208 cal BC/6238 ± 34 BP), and this specimen carried the European E1-C haplotype. Finally, the first appearance of the European E1-A haplotype in both group 1S and 2L pigs occurred during the Bronze Age at the site of Rotbav (Wietenberg culture; 2000–1500 cal BC; electronic supplementary material, table S3). This haplotype then became nearly ubiquitous in domestic pigs by 3900 BC, and remains so to this day [10].

**Figure 3.**
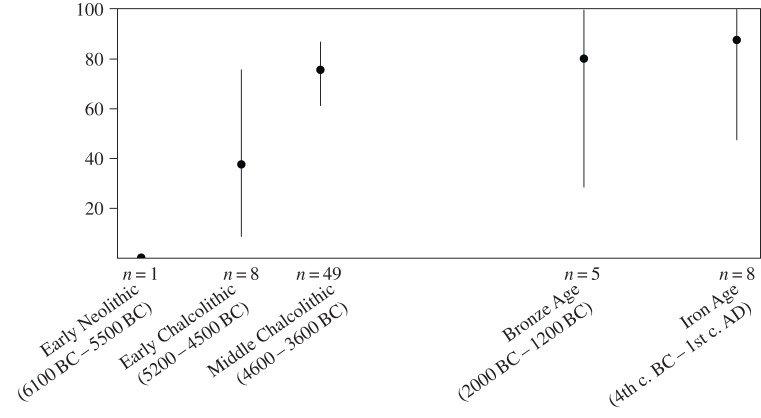
Evolution over time of the proportion of second and third molars belonging to the same individual showing matching wild or domestic identifications. Vertical bars correspond to the 90% confidence intervals of the percentages.

In all the analyses discussed above, each molar was analysed separately. However, because 71 mandibles and maxillae possessed both the second and third molars (electronic supplementary material, table S2), we were able to compare tooth shape identifications for each molar present in the same individual. Of the 71 specimens, only 50 possessed matching identifications for both molars. Of the 21 individuals with mismatched molar identifications, 15 pairs of teeth (including M_2_ and M_3_ or M^2^ and M^3^) possessed second molars with wild shape and third molars with domestic shape, whereas six specimens possessed the reverse pattern. In addition (though based on a small sample size), the proportion of matching molar shape identifications appears to increase through time before reaching 87.5% during the Iron Age (figure 3).

## 3. Discussion

For over 150 years, numerous theories regarding the geographical and temporal origins of pig domestication have been posited. In addition, the theoretical pathways to domestication and the complexity of the process have been widely discussed [12,39–44]. With regard to pigs, a great deal of debate has centred on whether the presence of domestic pigs in Neolithic contexts across Eurasia was the result of demic dispersal, cultural diffusion (domestication of local wild boar triggered by ideas brought with the expanding Neolithic cultures) or independent domestication. Resolving these issues through an analysis of the zooarchaeological record remains challenging. The data presented here demonstrate that the correlation between size alone and domestic status is far from 100%, a pattern also seen in a recent study from northern Germany [17]. These data imply a far more complex domestication history—one that could be interpreted in favour of local domestication and feralization, but more likely reflects a scenario of continuous gene flow between wild and domestic *S. scrofa*.

### (a) Wild, domestic or hybrid?

By combining the assessments of both size and shape, our results demonstrate the presence of individuals with large molars whose tooth shapes (counterintuitively) are associated with domestic pigs. This observation could reflect long-term gene flow between wild and domestic forms, which likely played a more significant role in the establishment of many early domestic animal populations than previously thought [12,13]. Although first-generation hybrids possess an equal genomic proportion from their wild and domestic ancestry, hybrid phenotypes may not necessarily appear intermediary between the two parents as a result of epistatic interactions (see references in [45]). As a result, a rapid decrease in size should be expected for first-generation hybrids between wild and domestic *S. scrofa*, but not necessarily in the case of continuous admixture between domestic pigs and local wild boar.

In the data presented here, the molars of the group 2L pigs that possess domestic tooth shapes are slightly smaller than those with a wild shape, suggesting that, in addition to altering their shape signatures, the process of becoming domestic (either independently or via admixture with introduced domestic populations) also influenced their size. If the earliest group 2L pigs with domestic tooth shape are in fact wild-domestic hybrids, they are probably not first-generation crosses, but more likely the result of multiple instances of (and perhaps even regular) introgression. However, we cannot exclude the possibility that these pigs were the result of an independent process that did not involve admixture with imported pigs, and future analyses of the nuclear genome should differentiate between these two scenarios.

### (b) Evidence for the tempo of phenotypic changes during domestication

Early animal domestication was most likely a multi-generational, progressive process. As a result, intermediate forms between wild and domestic phenotypes should be present in the archaeological record, at least during the earliest phases [42,46]. Although little is known about the differential impact and tempo domestication had on individual teeth, studies on murine rodents have demonstrated that size and shape along the molar tooth row vary significantly—the third molar varying the most, likely the result of a combination of genotypic, developmental and functional constraints [45].

Despite our small dataset, analyses of the 71 individuals that possessed both second and third molars hints at a differential tempo of shape change (at least on the two molars studied) as a result of domestication. The temporal pattern suggests a possible increase in the proportion of congruent molar identification through time. It also appears that the third molar reacts more quickly to the influence of domestication, an observation (based upon shape) that supports previous research on *S. scrofa* (based on size) [3,5]. This differential tempo of selection pressures on neighbouring teeth in the same jaw may reflect the intensity of domestication or hybridization, though this requires confirmation.

### (c) Were early domestic pigs in Romania introduced or the result of local domestication?

Although small pigs assumed to be domestic on the basis of postcranial measurements have previously been identified from the early Neolithic Starčevo Criş phase I of Măgura–Boldul lui Moş Ivănuş (6049–5915 cal BC/7107 ± 29 BP) [26], our small sample size meant we did not observe group 1S pigs in Early Neolithic contexts. The earliest specimen from group 1S was directly dated to 5318–5206 cal BC/6260 ± 34 BP, a Middle Neolithic context at Măgura–Buduiasca (Vădastra culture). Like the vast majority of Chalcolithic group 1S pigs, this individual carried the Y1 haplotype that was also ubiquitous in Neolithic and Chalcolithic pigs in western Anatolia [10,11].

Group 2L pigs with domestic tooth shape were identified in the Early Neolithic Măgura–Boldul lui Moş Ivănuş and at Cârcea La Hanuri. Although these failed to yield DNA, we obtained DNA from two Early Neolithic specimens (two wild boars identified using GMM and one young individual possessing only M1 and dp4, and therefore not included in GMM analysis), both of which carried the E1-C haplotype. This haplotype is identical to one identified in a group 2L pig, possessing a domestic tooth shape, from the Middle Neolithic, indicating its direct affinity with local wild boar (figure 2 and electronic supplementary material, figure S6). In fact, we observe a close genetic affinity between local wild boar and group 2L pigs possessing domestic tooth shape signatures across all time periods in which these groups coexisted (figure 2).

The combined GMM and aDNA data (electronic supplementary material, table S4) demonstrate that Neolithic–Chalcolithic group 1S pigs almost exclusively possessed the Y1 haplotype, suggesting genetic continuity between Near Eastern populations [11] and the early European domestic population in Romania. The Y1 haplotype was also observed in two Middle Chalcolithic specimens with large molars and domestic shape from the sites of Luncaviţa and Borduşani, something also previously reported from the site of Grube–Rosenhof in northeast Germany dated to the younger Ertebølle approximately 4750–4450 cal BC [17]. Lastly, two Middle Chalcolithic wild boar samples (from Borduşani) possessed the Y1 haplotype. Either this haplotype had a broader geographical range than previously thought, or, because all current data indicate an exclusive southwest Asian origin for the Y1 haplotype [9–11], its occurrence in group 2L specimens (with both domestic and wild tooth shape) is more likely the result of gene flow between introduced domestic pigs and local wild boar.

Finally, the earliest group 1S pigs with European mtDNA ancestry do not appear in Romania until the Bronze Age (Rotbav, Wietenberg culture). All these pigs possessed the E1-A haplotype, which in Romania was previously only observed in a Mesolithic specimen from the site of Schela Cladovei [10]. These data provide evidence for a domestic population turnover in Romania, similar and possibly linked to that previously reported from Anatolia and the Levant [11,47].

## 4. Conclusion

The data presented in this study demonstrate that though the majority of suids with large molars possessed a wild tooth shape (most likely representing wild boar), 40% possessed a domestic tooth shape. In addition, large individuals with domestic tooth shapes first appear during the early Romanian Neolithic and persist throughout later prehistory. Genetically, while the large pigs possess a variety of indigenous and imported haplotypes, the first small domestic pigs in Romania appeared no later than the Middle Neolithic and possessed a mitochondrial haplotype (Y1) identical to the one found in pigs from earlier Neolithic contexts in western Anatolia [11]. This haplotype was then replaced by an indigenous European mtDNA haplotype (E1-A) during the Bronze Age (2000–1200 cal BC).

The presence of a European haplotype in the large specimens with domestic shapes could be explained by local management leading to domestication (without any influence from the introduced [near eastern] domestics). The temporal pattern of the genetic and morphometric signatures, combined with the existence of individuals possessing molars with conflicting wild and domestic identifications, suggests that the most parsimonious explanation is not independent domestication of European wild boar, but instead, reciprocal gene flow between local wild populations and introduced domestic stocks.

By simultaneously investigating size, shape and mtDNA signatures in ancient pig specimens from the Mesolithic to Iron Age, our dataset has begun to reveal the shifting spatial and chronological complexity of phenotypic and genotypic signatures that resulted when non-endemic domestic pigs came into contact with the indigenous European wild boar. Subsequent analyses incorporating not just more samples, but also additional techniques including genome sequencing, will further reveal the increasingly complex narrative of domestication, human-assisted transport, gene flow and population replacement that has just begun to be deciphered.
